# Safety, resource use and nutritional content of home-blended diets in children who are gastrostomy fed: findings from ‘YourTube’ – a prospective cohort study

**DOI:** 10.1136/archdischild-2023-326393

**Published:** 2023-12-21

**Authors:** Lorna K Fraser, Andre Bedendo, Mark O’Neill, Jo Taylor, Julia Hackett, Karen Alice Horridge, Janet Cade, Gerry Richardson, Han Phung, Alison McCarter, Catherine Elizabeth Hewitt

**Affiliations:** 1 Cicely Saunders Institute, King's College London, London, UK; 2 Health Sciences, University of York, York, UK; 3 University of Sunderland, Sunderland, UK; 4 University of Leeds, Leeds, UK; 5 University of York Centre for Health Economics, York, UK; 6 Somerset Partnership NHS and Social Care Trust, Taunton, UK

**Keywords:** Child Health, Gastroenterology, Paediatrics

## Abstract

**Objective:**

To assess the risks, benefits and resource implications of using home-blended food in children with gastrostomy tubes compared with currently recommended formula feeds.

**Design:**

This is a cohort study. Data were collected at months 0, 12 and 18 from parents and clinicians using standardised measures.

**Setting:**

32 sites across England: 28 National Health Service trusts and 4 children’s hospices.

**Patients:**

Children aged 6 months–18 years who were gastrostomy fed.

**Main outcome measure:**

The main outcome measure was the PedsQL Gastrointestinal Symptoms Scales score. Secondary outcomes included quality of life, sleep (child, parent), dietary intake, anthropometry, healthcare usage, safety outcomes and resource use.

**Results:**

180 children and families completed the baseline data collection, with 134 (74%) and 105 (58%) providing follow-up data at 12 and 18 months. There were fewer gastrointestinal (GI) symptoms at all time points in the home-blended diet group, but there was no difference in change over time within or between the groups. The nutritional intake of those on a home-blended diet had higher calories per kilogram and fibre, and both home-blended and formula-fed children have values above the dietary reference values for most micronutrients. Safety outcomes were similar between groups and over time. The total costs to the statutory sector were higher among children who were formula fed, but the costs of purchasing special equipment for home-blended food and the total time spent on childcare were higher for families with home-blended diet.

**Conclusions:**

Children who are gastrostomy fed a home-blended diet have similar safety profile, adequate nutritional intake and lower burden of GI symptoms than formula-fed children.

**Trial registration number** ISRCTN13977361.

**WHAT IS ALREADY KNOWN ON THIS TOPIC**

- There is an increasing number of children who rely on gastrostomy feeds to meet their nutritional requirements.
- More parents are choosing to feed their children home-blended diets rather than the professional organisation recommendation of formula feeds.
- There is little evidence on the safety and nutritional intake of home-blended diets in these children compared with those receiving formula feeds.

**WHAT THIS STUDY ADDS**

- Children receiving a home-blended diet tend to come from less deprived areas and their parents have higher levels of education.
- Children receiving a home-blended diet have a similar safety profile to children receiving formula feeds.
- Home-blended diets have higher fibre intake and are associated with a lower burden of gastrointestinal symptoms.

**HOW THIS STUDY MIGHT AFFECT RESEARCH, PRACTICE OR POLICY**

- Home-blended diets for children who are gastrostomy fed should be seen as a safe alternative to formula feeding, unless there is a clinical contraindication.

## Introduction

The number of children who rely on gastrostomy tube feeding is rising, with the current prevalence estimated at 84 per 100 000 children or more than 10 000 children in England.^1^ Gastrostomy tube feeding is used when long-term reliance on enteral feeding due to unsafe swallow or food aversion is likely to be required. There are many underlying conditions associated with a requirement for enteral feeding, including neurological conditions, congenital cardiac disease, inherited metabolic conditions, cystic fibrosis, gastrointestinal conditions and cancer, with neurological conditions often the most common.^1^

In the UK, where access to healthcare is free at the point of care, commercially produced formula is recommended for children who are gastrostomy fed. There is, however, a growing number of parents who are choosing to feed their children home-blended diets in the UK,^2^ and this is also common in other countries.

Over the last decade, there have been concerns raised by professional bodies, including the European Society for Paediatric Gastroenterology Hepatology and Nutrition (ESPGHAN) and the British Dietetic Association (BDA), on potential safety issues with use of a home-blended diet, for example increased numbers of blocked gastrostomy tubes and gastrointestinal and stoma infections, concerns about the nutritional content of a home-blended diet, and questions about being able to meet the macronutrient and micronutrient requirements of children given this type of feed. At the start of this study, these professional bodies did not recommend use of home-blended diets to feed children with gastrostomies.^3 4^ However, during the period of this study, the position statements of these professional organisations, including the ESPGHAN^2 5^ and the BDA,^6^ moved to being more supportive, but still call for a more robust evidence on the safety and nutritional content of home-blended diets.

This study aimed to assess the risks, benefits and resource implications of using home-blended food in children with gastrostomy tubes compared with currently recommended formula feeds. This paper reports the 12-month and 18-month follow-up findings from this study.

## Materials and methods

This prospective cohort study was registered (ISRCTN13977361) and was conducted according to a published protocol.^7^ The COVID-19 pandemic impacted on the recruitment to this study when all clinical research was paused within the National Health Service (NHS) in the UK in early 2020. This resulted in a reduction in the target sample size (from 300 to 180) and a change in data collection schedules from 0, 9 and 18 months to 0, 12 and 18 months.

Children and parents were recruited through 31 NHS sites from August 2019 until November 2021. Data were collected at baseline and at 12 and 18 months, with the final data collection completing on 31 May 2023.

All children aged 6 months–<19 years who received all or part of their nutrition via a gastrostomy tube were eligible to be included in this study. Data were collected on paper or via online survey tools from parents, clinicians, and where appropriate the children or the young people themselves. Nutritional intake data were collected using published data for formula feeds and using myfood24^8^ for those with a home-blended diet. Anthropometric data were collected from clinician or parent report, with the COVID-19 pandemic increasing the parent-reported data. A short video was available for professionals and parents to measure the mid-upper arm circumference (MUAC; see https://www.york.ac.uk/healthsciences/research/public-health/projects/yourtube/studyresources/).

Detailed information on all data collection is available in the protocol^7^ and baseline publication.^9^ Information on resource use and costs was also collected from the parents at all time points.

### Analyses

#### Statistical analyses

All statistical analyses were undertaken using R and an alpha of 5%. Descriptive statistics for all clinical, demographic and outcome information used mean, SD and 95% CI for continuous data, and counts and percentages for categorical data. The primary outcome was the PedsQL Gastrointestinal Symptoms Scales score.^10^ The secondary outcomes were children’s quality of life (DISABKIDS Short Form, EuroQol-5 Dimension Visual Analogue Scale and the five-component scale of the five-level version of EuroQol-5 Dimension (EQ-5D-5L)),^11^ Parenting Morale Index,^12^ child (parent report) and parental sleep (Patient-Reported Outcomes Measurement Information System, or PROMIS), Body Mass Index Standard Deviation Score (BMISDS), MUAC, nutritional intake (total kilocalories, kilocalories per kilogram and % of energy from macronutrients considering the dietary reference value (DRV)), macronutrients (protein, carbohydrate, fat and Association of Analytical Chemists (AOAC) fibre) and micronutrients (vitamin B_12_, vitamin D, folate, calcium, iron, manganese, zinc), and safety outcomes (number of children reporting and the number of occurrences of gut-intestinal infection, stoma site infection and tube blockage; gastrostomy tube needing replacement; pneumonia; and accident and emergency (A&E) attendances in the last 12 months and the number of children reporting any and the number of occurrences).

Graphical summaries were used to show trends in primary and secondary outcomes over time. When appropriate, group comparisons used analysis of variance and Pearson’s χ^2^ tests. Summaries were provided overall and by the two groups of interest using baseline allocation: those who are 100% formula fed and those with any amount of home-blended feeds.

Propensity scores were used to balance the sample for demographic baseline data^13 14^ using the Index of Multiple Deprivation score and calculated using package *WeightIt* V.0.13.1. The propensity score weights were applied in a generalised linear mixed model (GLMM) using the PedsQL total score measured at baseline as the outcome; group, age, sex and diagnosis as fixed effects; and recruitment site as a random effect. Assumptions were checked using graphical and GLMM inspection of Akaike information criterion values. Inferential analyses were not performed on secondary outcomes due to the large amount of outcome data collected and concerns over multiple testing.

#### Health economic analyses

The objective of the health economic evaluation was to assess the costs, resource use and the associated health-related quality of life of providing a home-blended diet compared with a formula-fed diet only to children with gastrostomy tubes. We compared the costs of providing formula and healthcare under an NHS and personal and social services (PSS) perspective. To calculate the total cost for each child, a micro-costing framework was used. A multiple imputation model was employed with the number of chains were considered using a two-step approach.^15^ The unit cost of formula food was acquired from the British National Formulary website,^16^ while the unit cost for health and social care services was derived from Personal Social Services Research Unit (PSSRU) 2021.^15^ The cost of equipment purchased exclusively for home-blended diet and the total time associated with childcare, categorised into time spent on preparing and administering food, time spent on preparing and administering medications, and time spent on caring for gastrostomy, were also collected and provided using complete case analysis.

### Patient and public involvement

Parents whose children were gastrostomy fed (n=7) were involved in the development and management of this study. They prioritised outcomes, helped develop appropriate recruitment methods, including the use of social media, and contributed to the study materials. They also chose the study title ‘YourTube’ and are actively involved in interpreting the study findings and dissemination.

## Results

180 children and families completed the baseline data collection, with 134 (74%) and 105 (58%) providing follow-up data at 12 and 18 months, respectively (online supplemental figure 1).

10.1136/archdischild-2023-326393.supp1 Supplementary data

The clinical and demographic information at baseline and at 12 and 18 months (table 1) shows that while the two groups were similar in terms of age, sex and underlying diagnoses, the children who were receiving a home-blended diet tended to come from areas of lower deprivation and have parents with higher levels of education. Children from areas with higher levels of deprivation were less likely to complete the study and the parents who continued with the study had higher educational qualifications.

**Table 1:** Clinical and demographic characteristics of the cohort at baseline and at 12-month and 18-month follow-up*

	Baseline (n=180)		12 months (n=134)		18 months (n=105)	
	Home-blended (n=104)	Formula fed (n=76)	Home-blended (n=79)	Formula fed (n=55)	Home-blended (n=57)	Formula fed (n=48)
Age (years)						
Mean (SD)	9.2 (4.4)	10.2 (4.4)	9.2 (4.4)	10.4 (4.6)	9.4 (4.4)	10.5 (4.8)
Sex, n (%)						
Female	38 (37.3)	32 (42.7)	28 (36.4)	22 (40.7)	22 (40.0)	19 (39.6)
Male	64 (62.7)	43 (57.3)	49 (63.6)	32 (59.3)	33 (60.0)	29 (60.4)
Missing	2	1	2	1	2	–
Index of Multiple Deprivation, n (%)						
1 (most deprived)	15 (14.4)	17 (22.4)	8 (10.1)	8 (14.5)	5 (8.8)	5 (10.4)
2	18 (17.3)	18 (23.7)	16 (20.3)	15 (27.3)	10 (17.5)	12 (25.0)
3	18 (17.3)	16 (21.1)	16 (20.3)	15 (27.3)	13 (22.8)	14 (29.2)
4	27 (26.0)	18 (23.7)	18 (22.8)	11 (20.0)	14 (24.6)	11 (22.9)
5 (least deprived)	26 (25.0)	7 (9.2)	21 (26.6)	6 (10.9)	15 (26.3)	6 (12.5)
Parental educational qualification, n (%)						
School leaving qualifications	13 (12.7)	20 (26.7)	10 (13.0)	14 (25.5)	10 (18.2)	12 (25.0)
Further education	21 (20.6)	26 (34.2)	15 (19.5)	17 (30.9)	10 (18.2)	15 (31.2)
Higher education	67 (65.7)	26 (34.7)	52 (67.5)	21 (38.2)	35 (63.6)	20 (41.7)
Other/no educational qualifications	1 (1.0)	4 (5.3)	0 (0.0)	3 (5.5)	0 (0.0)	1 (2.1)
Missing	2	1	2	–	2	–
Child’s ethnicity, n (%)						
White British	88 (88.9)	60 (81.1)	71 (89.9)	44 (80.0)	50 (87.7)	39 (81.2)
Other	11 (11.1)	14 (18.9)	8 (10.1)	11 (20.0)	7 (12.3)	9 (18.8)
Missing	24	10	–	–	–	–
Children’s diagnostic group, n (%)						
Neurological	43 (41.7)	25 (32.9)	33 (42.3)	22 (40.0)	23 (41.1)	20 (41.7)
Genetic	41 (39.8)	33 (43.4)	32 (41.0)	23 (41.8)	23 (41.1)	19 (39.6)
Congenital	11 (10.7)	10 (13.2)	9 (11.5)	6 (10.9)	7 (12.5)	6 (12.5)
Other	8 (7.8)	8 (10.5)	4 (5.1)	4 (7.3)	3 (5.4)	3 (6.2)
Missing		0				
Gastrostomy type, n (%)						
Button (Mini or Mic-Key)	89 (86.4)	64 (84.2)	65 (83.3)	47 (85.5)	48 (85.7)	42 (87.5)
PEG	12 (11.7)	7 (9.2)	11 (14.1)	4 (7.3)	8 (14.3)	4 (8.3)
Other	2 (1.9)	5 (6.6)	2 (2.6)	4 (7.3)	0 (0.0)	2 (4.2)
Missing	1	0				
Gastrostomy duration in years						
Mean (SD)	5.6 (3.9)	7.2 (4.7)	5.7 (3.9)	7.6 (4.9)	6.2 (3.9)	7.7 (5.1)
Range	0.0–15.0	0.0–18.0				
Missing	5	1				
Fundoplication, n (%)						
No	72 (69.2)	41 (54.7)	52 (65.8)	30 (55.6)	37 (64.9)	23 (48.9)
Yes	32 (30.8)	34 (45.3)	27 (34.2)	24 (44.4)	20 (35.1)	24 (51.1)
Missing	0	1				

*Some missing clinical and demographic data from baseline were updated using 12-month and 18-month data if available.

PEG, percutaneous endoscopic gastrostomy.

### Primary outcome

**Figure 1:**
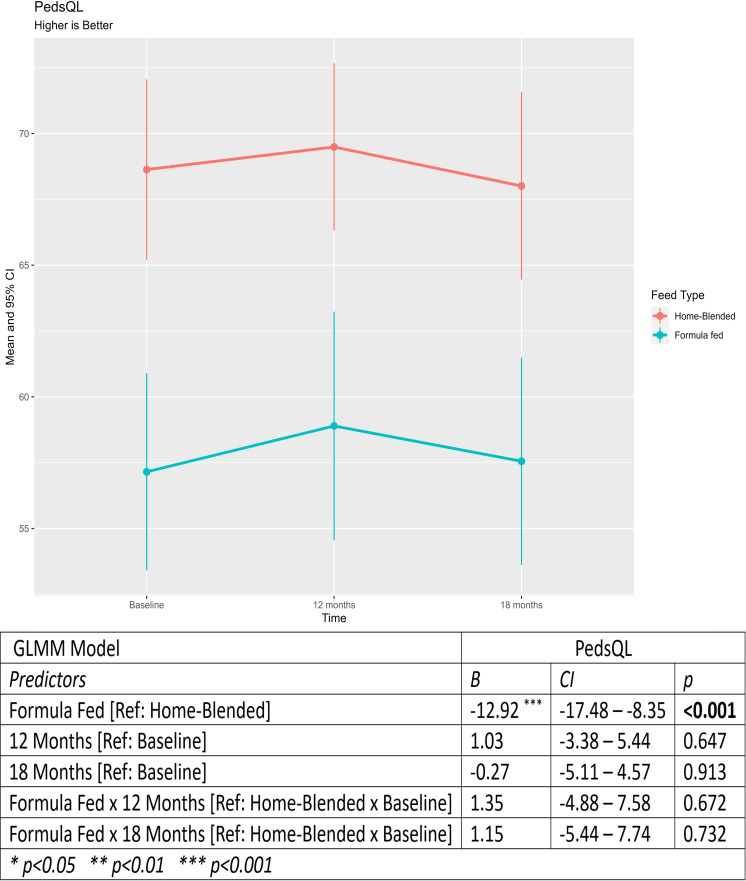
Primary outcome: PedsQL Gastrointestinal Symptoms Scale score at baseline and at 12 and 18 months. GLMM, generalised linear mixed model.

Figure 1 shows the change over time in the primary outcome, PedsQL Gastrointestinal Symptoms Scales score. The results from the GLMM model (figure 1) showed that overall the formula-fed group had more gastrointestinal symptoms than the home-blended group; however, there was no significant effect of time, and the change over time was not different between groups.

### Nutritional content

Information was available for ~86% of the children at baseline and at 12 and 18 months (table 2). Those missing nutritional data were all in the home-blended group.

**Table 2:** Nutritional content at baseline and at 12-month and 18-month follow-up

	Baseline (n=180)		12 months (n=134)		18 months (n=105)	
	Home-blended (n=104)	Formula fed (n=76)	Home-blended (n=79)	Formula fed (n=55)	Home-blended (n=57)	Formula fed (n=48)
Macronutrients						
Kilocalories per kilogram	61.3 (54.1, 68.5)	44.0 (38.9, 49.1)	60.7 (51.6, 69.8)	41.0 (35.7, 46.2)	63.4 (50.9, 75.8)	40.9 (35.1, 46.7)
Total kilocalories	1231.2 (1107.6, 1354.8)	1114.2 (1009.1, 1219.2)	1351.1 (1186.2, 1516.0)	1154.5 (1034.7, 1274.2)	1443.8 (1239.1, 1648.5)	1172.6 (1036.4, 1308.7)
% kilocalories from protein	14.3 (13.3, 15.3)	12.8 (12.0, 13.7)	14.7 (13.4, 16.1)	13.8 (12.7, 15.0)	14.3 (13.2, 15.4)	13.7 (12.5, 14.9)
% kilocalories from carbohydrate	45.5 (43.1, 47.8)	47.9 (47.2, 48.6)	43.9 (41.4, 46.4)	47.6 (45.5, 49.7)	43.1 (40.0, 46.2)	48.1 (45.6, 50.6)
% kilocalories from fat	40.2 (37.5, 42.8)	38.0 (36.9, 39.1)	41.4 (38.5, 44.2)	37.5 (35.0, 40.0)	42.4 (38.8, 45.9)	37.2 (34.4, 40.1)
Total amount						
Carbohydrate (g)	139.3 (123.6, 154.9)	134.8 (121.3, 148.3)	145.2 (127.8, 162.6)	138.4 (121.9, 154.9)	151.5 (132.9, 170.1)	142.1 (122.9, 161.4)
Protein (g)	44.0 (39.0, 48.9)	35.9 (31.4, 40.4)	49.1 (42.7, 55.4)	39.7 (34.2, 45.3)	50.7 (43.4, 58.0)	40.2 (33.9, 46.6)
Fat (g)	55.6 (48.9, 62.3)	46.3 (42.3, 50.4)	64.0 (52.9, 75.0)	47.8 (42.4, 53.2)	70.6 (55.8, 85.4)	48.0 (41.9, 54.1)
Fibre (g)	14.1 (11.8, 16.4)	6.3 (4.9, 7.7)	14.5 (11.7, 17.4)	6.9 (4.9, 9.0)	16.5 (13.2, 19.7)	7.2 (5.0, 9.4)
Grams per kilogram						
Carbohydrate (g/kg)	7.0 (6.0, 7.9)	5.3 (4.7, 5.9)	6.6 (5.6, 7.6)	4.8 (4.2, 5.3)	6.6 (5.5, 7.7)	4.8 (4.1, 5.4)
Protein (g/kg)	2.2 (1.9, 2.5)	1.3 (1.2, 1.5)	2.2 (1.9, 2.5)	1.3 (1.2, 1.5)	2.2 (1.8, 2.6)	1.3 (1.2, 1.5)
Fat (g/kg)	2.8 (2.4, 3.1)	1.9 (1.6, 2.1)	2.9 (2.3, 3.5)	1.8 (1.4, 2.2)	3.2 (2.3, 4.0)	1.8 (1.3, 2.3)
Kilocalories (%DRV)	76.5 (67.7, 85.3)	61.9 (55.5, 68.3)	83.5 (71.7, 95.2)	64.6 (57.0, 72.2)	88.8 (72.8, 104.8)	66.0 (57.7, 74.2)
Micronutrients						
B_12_ (%DRV)*	284.9 (235.8, 333.9)	253.4 (221.9, 284.8)	313.9 (253.6, 374.2)	265.5 (234.3, 296.7)	369.6 (237.1, 502.0)	278.1 (242.1, 314.1)
Folate (%DRV)	151.7 (131.3, 172.0)	242.0 (216.6, 267.3)	164.0 (141.2, 186.8)	249.8 (220.7, 279.0)	157.4 (129.7, 185.1)	256.6 (223.8, 289.5)
Vitamin D (%DRV)	55.2 (44.2, 66.2)	120.4 (107.1, 133.8)	55.2 (43.6, 66.9)	124.9 (112.2, 137.7)	55.8 (40.4, 71.1)	130.2 (114.8, 145.7)
Calcium (%DRV)	125.5 (110.1, 140.8)	144.6 (120.8, 168.5)	139.3 (120.1, 158.5)	165.2 (129.3, 201.2)	138.8 (117.1, 160.6)	171.4 (132.0, 210.9)
Iron (%DRV)	120.1 (104.5, 135.8)	136.0 (122.8, 149.1)	129.3 (113.2, 145.4)	147.5 (134.3, 160.7)	126.3 (106.8, 145.7)	152.4 (134.9, 169.9)
Manganese (%DRV)	136.0 (112.7, 159.2)	120.1 (98.8, 141.4)	134.6 (114.1, 155.2)	141.2 (112.0, 170.5)	157.5 (125.5, 189.4)	145.0 (113.0, 177.0)
Zinc (%DRV)	124.0 (106.9, 141.2)	211.8 (176.9, 246.6)	143.0 (118.7, 167.3)	221.1 (183.9, 258.3)	134.8 (109.1, 160.5)	220.3 (180.3, 260.3)
Missing	19	5	16	3	13	1

*Missing at baseline: 26; at 12 months: 19; at 18 months: 15. Additional missing due to inconsistent data (eg, extreme values).

DRV, dietary reference value.

The patterns of nutritional intake in the two groups were similar across time:

- Macronutrient content: The fibre intake was higher in the home-blended group. The kilocalorie intake per kilogram of bodyweight was higher in the home-blended diet group, with similar per cent of diet in both groups from fat, protein and carbohydrate.
- Micronutrient content: Both the home-blended and formula-fed children had values above the DRV for vitamin B_12_, folate, vitamin D, calcium, iron, manganese and zinc. Only vitamin D was insufficient in the home-blended group.

### Anthropometry

The BMISDS and MUAC were highly variable within the groups, but the mean BMISDS was in the normal range in both groups and across time (online supplemental figure 2).

### Safety outcomes

The mean numbers of proximal safety outcomes were similar between the groups and over time, that is, the number of gastrostomy tube replacements (table 3): home-blended versus formula fed: 3.0 vs 3.4, 3.2 vs 3.2, and 1.8 vs 1.5 at 0, 12 and 18 months. The mean number of visits to A&E and episodes of pneumonia was also similar between the groups; gut and stoma infections were not significantly different between the groups at baseline and at 12 months (table 3).

**Table 3:** Safety outcomes at baseline and at 12-month and 18-month follow-up

	Home-blended: any follow-up (n=104)	Formula fed: any follow-up (n=76)	Home-blended: 12 months (n=79)	Formula fed: 12 months (n=55)	Home-blended: 18 months (n=57)	Formula fed: 18 months (n=48)
Number of gut infections during the last 12 months						
Mean (95% CI)	1.5 (0.9, 2.1)	2.7 (1.5, 3.9)	1.1 (0.8, 1.4)	2.4 (0.7, 4.2)	1.3 (−0.1, 2.8)	1.2 (0.5, 2.0)
Number of stoma site infections during the last 12 months						
Mean (95% CI)	1.3 (0.8, 1.9)	3.0 (1.8, 4.2)	1.5 (−0.1, 3.1)	2.1 (1.0, 3.2)	1.2 (0.7, 1.8)	1.9 (0.6, 3.2)
Number of tube blockages during the last 12 months						
Mean (95% CI)	2.9 (2.0, 3.8)	4.7 (−0.1, 9.5)	3.0 (1.7, 4.2)	6.7 (−6.9, 20.3)	1.8 (0.9, 2.7)	2.0 (−0.3, 4.3)
Number of replacements during the last 12 months						
Mean (95% CI)	3.0 (2.6, 3.4)	3.4 (2.9, 3.8)	3.2 (2.8, 3.7)	3.2 (2.8, 3.7)	1.8 (1.5, 2.1)	1.5 (1.3, 1.7)
Number of times with pneumonia during the last 12 months						
Mean (95% CI)	1.6 (1.2, 2.1)	3.2 (−0.0, 6.4)	1.7 (1.1, 2.2)	1.1 (0.8, 1.5)	1.4 (0.8, 2.0)	1.5 (0.6, 2.4)
Number of visits to A&E						
Mean (95% CI)	1.1 (0.7, 1.5)	1.4 (0.7, 2.1)	0.9 (0.5, 1.3)	0.9 (0.5, 1.2)	0.5 (0.3, 0.7)	0.6 (0.3, 0.8)

A&E, accident and emergency.

### Health economic outcomes

The total costs to the NHS and PSS, comprising the cost of formula food and the cost of health and social care services, were higher among children with formula-fed diet than those with home-blended diet: £16 386 vs £12 028 per annum at baseline, £18 049 vs £14 357 per annum at 12 months, and £8345 vs £5887 per half-year at 18 months, respectively (see tables 4 and 5). The cost of formula food mainly contributed to such differences. As a trade-off, families in home-blended group spent an estimated £294 in the previous 12 months (at baseline), £176 in the previous 12 months (at 12 months) and £97 in the previous 6 months (at 18 months) on kitchen equipment for blending and storing blended food. They also spent an average of 88 min (at baseline), 85 min (at 12 months) and 103 min (at 18 months) higher per day caring for children than those in the formula-fed group.

**Table 4:** Health and social care resource use at baseline and at 12-month and 18-month follow-up

	Baseline*		12 months*		18 months†	
Health and social care resource use, mean (SD)	Home-blended (n=104)	Formula fed (n=76)	Home-blended (n=79)	Formula fed (n=55)	Home-blended (n=57)	Formula fed (n=48)
General practitioner visit	0.54 (0.50)	0.47 (0.50)	2.32 (9.05)	1.11 (1.33)	1.00 (1.54)	1.08 (1.54)
Paediatrician visit	2.66 (2.50)	2.96 (4.85)	2.46 (3.23)	2.05 (1.69)	1.18 (0.95)	1.15 (1.05)
Speech and language therapist visit	5.40 (11.30)	2.93 (6.44)	5.05 (9.52)	5.09 (11.02)	2.70 (5.77)	3.42 (8.84)
Physiotherapist visit	11.47 (20.63)	6.13 (11.82)	10.14 (14.08)	7.04 (10.74)	5.77 (7.61)	4.23 (7.87)
Community children nurse team visit	6.42 (9.50)	6.03 (8.75)	5.63 (10.54)	5.67 (7.87)	2.63 (6.96)	3.31 (7.82)
Dietitian visit	3.58 (3.51)	3.32 (2.94)	2.99 (2.99)	2.95 (1.87)	1.28 (1.19)	1.71 (1.56)
Hospital night stay	5.13 (10.09)	5.95 (12.38)	8.63 (31.96)	6.98 (17.41)	1.63 (3.67)	7.44 (20.00)
A&E visit	1.26 (2.17)	1.36 (3.08)	0.92 (1.72)	0.87 (1.26)	0.51 (0.87)	0.63 (0.94)

*Annual resource use.

†Semiannual resource use.

A&E, accident and emergency.

**Table 5:** Total cost per gastrostomy-fed child under the PSS and NHS perspective (£, 2021)

	Baseline*		12 months*		18 months†	
Cost component, mean (SD)	Home-blended (n=104)	Formula fed (n=76)	Home-blended (n=79)	Formula fed (n=55)	Home-blended (n=57)	Formula fed (n=48)
Formula food	2315 (3679)	6485 (4770)	2489 (4528)	8016 (7291)	1070 (1414)	3429 (3496)
Health and social care services	9713 (11 688)	9901 (15 163)	12 047 (30 473)	10 033 (16 460)	3747 (4003)	4550 (9438)
Total	12 028 (13 307)	16 386 (15 704)	14 537 (31 495)	18 049 (18 132)	5887 (5447)	8345 (10 971)

*Annual cost.

†Semiannual cost.

NHS, National Health Service.

While the overall EQ-5D-5L scores in both groups were similar, more parents reported health issues on dimensions such as pain/discomfort and anxiety/depression at all time points (see online supplemental table 1 and online supplemental figure 3). The data on the other secondary outcomes are shown in online supplemental table and figures.

## Discussion

This large, prospective, national cohort study of children who were gastrostomy fed has shown that those who were gastrostomy fed a home-blended diet had similar safety profile, adequate nutritional intake and lower burden of gastrointestinal symptoms compared with formula-fed children. The home-blended diet was associated with lower costs for the statutory sector, but came with increased expenses for the families with equipment costs and childcare time, along with a small home-made food cost. The health-related quality of life outcomes for parents and children were similar between the two groups.

The lower burden of gastrointestinal symptoms in the home-blended diet group was maintained across the time period of this study, and these findings are consistent with the small number of published studies that have reported gastrointestinal symptoms.^17^

Children who required gastrostomy feeds are often fragile and at risk of recurrent infections, and concerns over the additional risk of using a home-blended diet have been discussed.^5^ In this study, there was no evidence of an increase in the number of stoma site, gut infections or pneumonia in the home-blended diet group compared with the formula-fed group.

One of the main concerns raised by professionals about the nutritional adequacy of home-blended diets relates to the viscosity of the feeds required to get through the tubes and therefore the large volumes that may be required to maintain an adequate calorific intake.^5 18^ In this study, the calorific intake was higher in the home-blended group across the time period while maintaining adequate anthropometric measures. Previous research has shown that gastrostomy-fed children may be able to tolerate higher volumes of home-blended feeds than formula.^18^ The micronutrient content in this current study was also relatively stable and above DRV for all, apart from vitamin D, which is similar to what cross-sectional studies have shown.^5 13^ Children who require specific nutritional content in their diet, for example ketogenic diets, may require more input from dietitians.

The economic analyses have shown that while using a home-blended diet is associated with a reduction in costs to the NHS and PSS, there is an associated increase in costs and time on care to families. This may in part explain why families in this study who used a home-blended diet tended to be from areas of lower deprivation. There is no financial cost to the family of formula feeds in the UK, but the distribution of costs may be different in other healthcare systems. The additional costs and time required to use a home-blended diet may mean that this is not a viable option for some families. Although there is an increase in the number of commercial companies producing prepackaged blended food, further research is required in terms of the impact of symptoms from prepackaged blended foods. Children with complex disabilities are already at risk of inequalities in access to health and social care, so future services and policies relating to enteral feeding must address the potential financial impact of a home-blended diet. The safety profile of home-blended diets should be useful to inform policies in schools and hospitals where parents report varying levels of support for the use of home-blended diets.^19^

### Strengths and limitations

This has been the largest study of home-blended diets in children with gastrostomies to date^17^ and the study used parent-prioritised outcomes. Study retention was good despite the impact of the pandemic; however, more of the home-blended group did come from areas of lower deprivation and the parents had higher levels of education. As this is an observational cohort study and not a randomised controlled trial, there should be caution over the causal implications. Consent for long-term follow-up using routine data was obtained from the participants of this study. Hospital use and survival are the main outcomes possible using this follow-up.

## Conclusions

Children who were fed a home-blended diet maintained an adequate nutritional intake and had no increase in safety events when compared with children who were formula fed in this 18-month study. There was an increase in financial costs to families of feeding a home-blended diet, and given the evidence that families who used a home-blended diet have higher levels of education and live in areas of lower deprivation, future policies should address inequalities in access. Home-blended diets should be seen as a safe alternative to commercial formula, unless there is a specific clinical contraindication. These data show that home-blended diets can provide similar nutritional intake to commercial formula in children who require gastrostomy feeding. High-quality studies are required to address any differences in long-term outcomes for children who are fed home-blended diets.

## Data Availability

Data are available upon reasonable request. Data may be available upon request to the PI if covered in the REC approval.
