# Supplementary material for: Safety, resource use and nutritional content of home-blended diets in children who are gastrostomy fed: findings from ‘YourTube’ – a prospective cohort study
Source: Arch Dis Child. 2023 Dec 21;109(8):628–35. doi: 10.1136/archdischild-2023-326393 (PMC11287525; doi:10.1136/archdischild-2023-326393)

Supplementary Tables and Figures

Supplementary Table 1 – Secondary Outcomes at baseline, 12 and 18 months

	Baseline n=180		12 months n=134		18 months n=105	
	Home-Blended - Any Follow Up (N=104)	Formula fed - Any Follow Up (N=76)	Home-Blended - 12 months (N=79)	Formula fed - 12 months (N=55)	Home-Blended - 18 months (N=57)	Formula fed - 18 months (N=48)
DISABKIDS - Quality of Life with Chronic Conditions						
M(95%CI)	57.1 (52.5, 61.7)	53.2 (48.2, 58.1)	51.7 (46.2, 57.1)	54.4 (47.5, 61.4)	56.0 (50.5, 61.4)	53.4 (46.9, 59.8)
Child Sleep Disturbance (PROMIS)						
M(95%CI)	61.1 (59.3, 62.8)	61.9 (59.9, 63.9)	59.9 (57.8, 62.1)	60.7 (58.5, 62.9)	59.2 (56.8, 61.6)	60.6 (58.1, 63.1)
EQ5D VAS - How good is the health of your child TODAY						
M(95%CI)	76.9 (73.1, 80.7)	71.6 (66.5, 76.7)	78.3 (74.1, 82.5)	73.2 (68.1, 78.4)	79.2 (75.0, 83.4)	75.0 (69.9, 80.2)
Mid-upper arm circumference (mm)						
M(95%CI)	196.8 (187.9, 205.7)	206.7 (196.4, 217.1)	204.8 (194.8, 214.7)	209.7 (197.5, 222.0)	205.1 (192.0, 218.2)	219.0 (205.3, 232.6)
Body Mass Index standard deviation score						
M(95%CI)	-0.4 (-0.8, 0.0)	0.2 (-0.2, 0.5)	-0.1 (-0.5, 0.4)	0.2 (-0.2, 0.6)	-0.6 (-1.3, 0.1)	-0.2 (-0.8, 0.5)
EQ5D VAS - How good is your health TODAY - Parent						
M(95%CI)	84.0 (81.0, 86.9)	79.6 (75.9, 83.3)	82.4 (79.1, 85.7)	78.3 (73.7, 82.9)	82.1 (78.5, 85.6)	78.6 (73.8, 83.3)
Parenting Morale Index						
M(95%CI)	29.0 (27.6, 30.4)	28.3 (26.5, 30.1)	29.3 (27.5, 31.1)	27.8 (26.0, 29.7)	28.5 (26.6, 30.4)	27.6 (25.3, 29.9)
EQ5D-5L - EuroQol Quality of Life - Parents						
M(95%CI)	0.7 (0.7, 0.8)	0.7 (0.7, 0.8)	0.8 (0.7, 0.8)	0.7 (0.7, 0.8)	0.8 (0.7, 0.8)	0.7 (0.7, 0.8)

Supplementary Figure 1 – Recruitment and retention

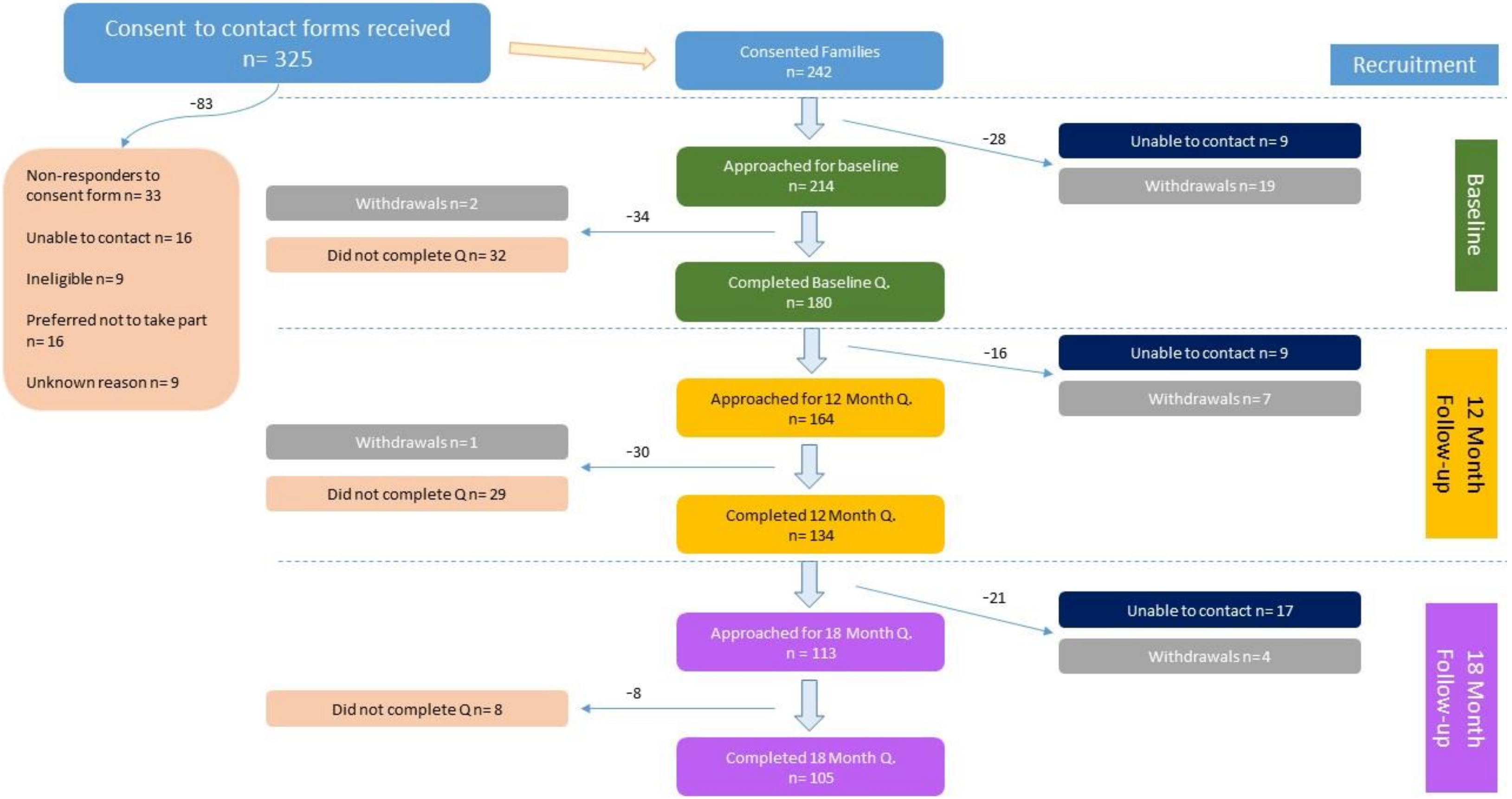

Supplementary Figure 2 Change in Secondary Outcomes at baseline, 12 and 18 months*

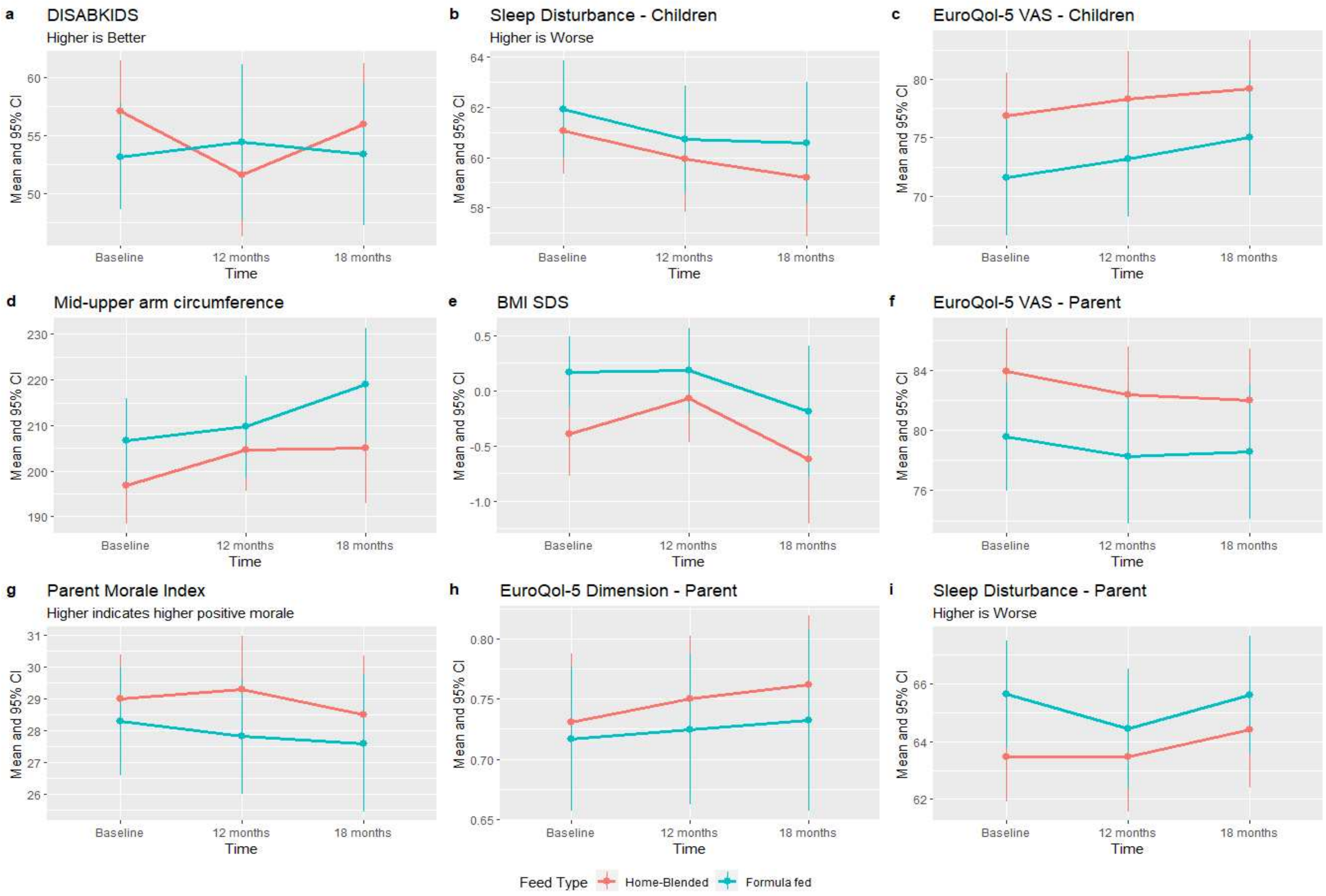

* N=180 at Baseline; N=134 at 12 months, N = 105 at 18 months

Supplementary Figure 3 Proportion of parents reporting a health problem

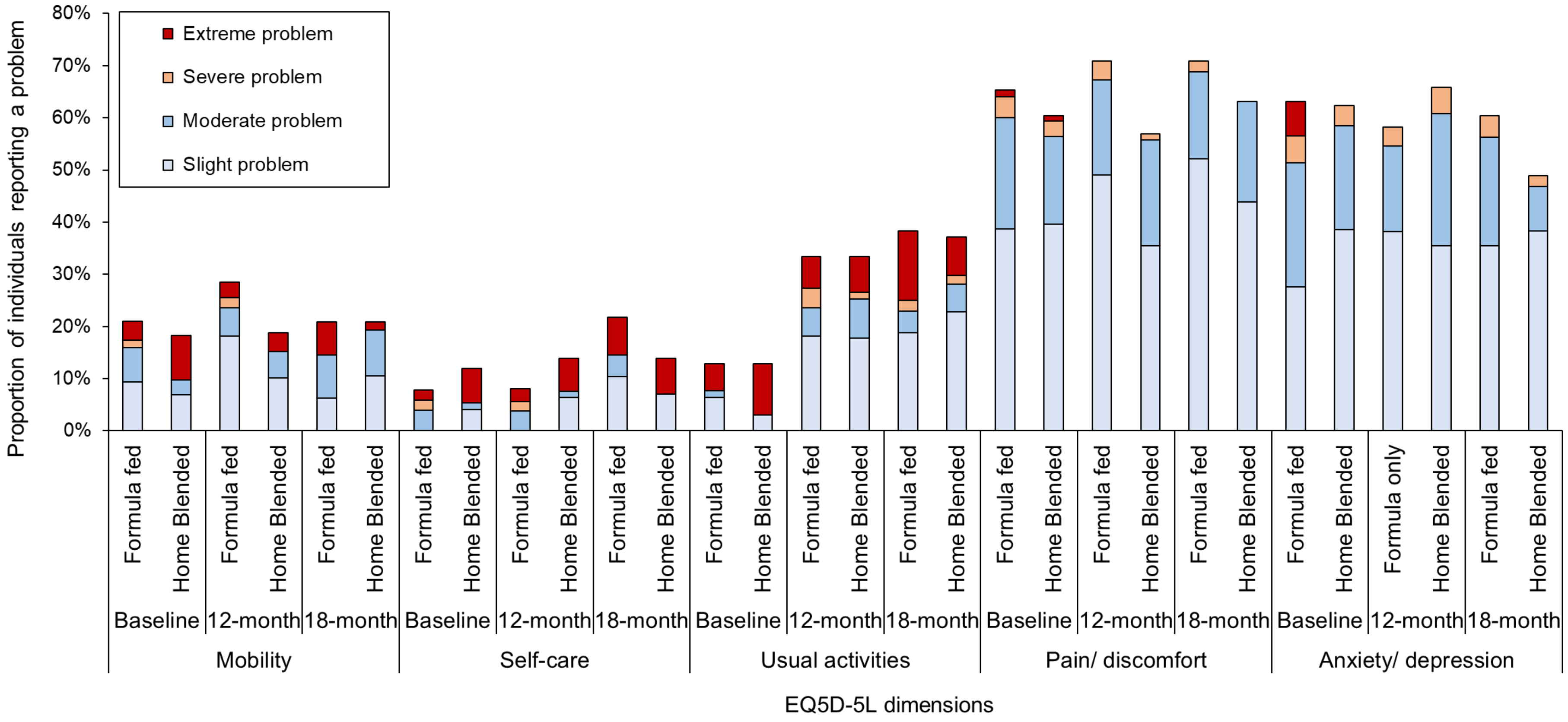

Supplement: Supplementary data [file archdischild-2023-326393supp001.pdf]
